# Vocal cord granuloma after transoral thyroidectomy using oral endotracheal intubation: two case reports

**DOI:** 10.1186/s12871-021-01393-8

**Published:** 2021-06-14

**Authors:** Tsung-Jung Liang, Nai-Yu Wang, Shiuh-Inn Liu, I-Shu Chen

**Affiliations:** 1 Division of General Surgery, Department of Surgery, Kaohsiung Veterans General Hospital, No. 386, Dazhong 1st Rd, Zuoying District, 81362 Kaohsiung, Taiwan; 2 School of Medicine, National Yang-Ming University, No.155, Sec.2, Linong Street, 11221 Taipei, Taiwan

**Keywords:** Vocal cord granuloma, Endoscopic thyroidectomy, Transoral, Vestibular approach, Thyroid

## Abstract

**Background:**

Transoral thyroidectomy can be performed using nasal or oral intubation. Recently, we encountered two cases of vocal cord granuloma that were suspected to result from intraoperative compression by the oral endotracheal tube.

**Cases presentation:**

Two women underwent transoral endoscopic thyroidectomy with oral endotracheal tubes fixed at the mouth angle. Their initial postoperative recovery was uneventful, but they developed hoarseness 2 months after the surgery. Subsequent strobolaryngoscopy revealed vocal cord granulomas at the side of contact of the endotracheal tube. One patient received medication and voice therapy, and her granuloma shrank significantly one month later. The other patient underwent granuloma resection. Thereafter, the symptoms improved in both the patients.

**Conclusions:**

Oral intubation with tube placement at the mouth angle might result in the formation of vocal cord granulomas. Therefore, we suggest positioning the tube at the midline to avoid excessive irritation on one side of the vocal cord.

## Background

Transoral thyroidectomy via the vestibular approach is a scar-free surgery that provides excellent cosmetic results while yielding surgical outcomes equivalent to those of traditional open surgery [1]. This approach uses three trocars placed in the oral vestibular area to perform thyroidectomy. However, the endoscopic instruments sometimes collide with the camera owing to the limited space in the oral cavity. To avoid this overcrowding, nasal intubation, instead of oral intubation, was used in the initial design of the surgical procedure described by Anuwong [2].

Nevertheless, nasal intubation might cause epistaxis, especially in patients with a narrow nasal cavity or deviated nasal septum, which might further impede the passage of the endotracheal tube and increase the risk of nasal injury [3]. Endotracheal tube compression might also result in the formation of pressure sores in the nasal ala [4]. Therefore, some surgeons attempted using oral intubation and found it to be a feasible alternative while performing transoral thyroidectomy [5–9].

**Fig. 1:**
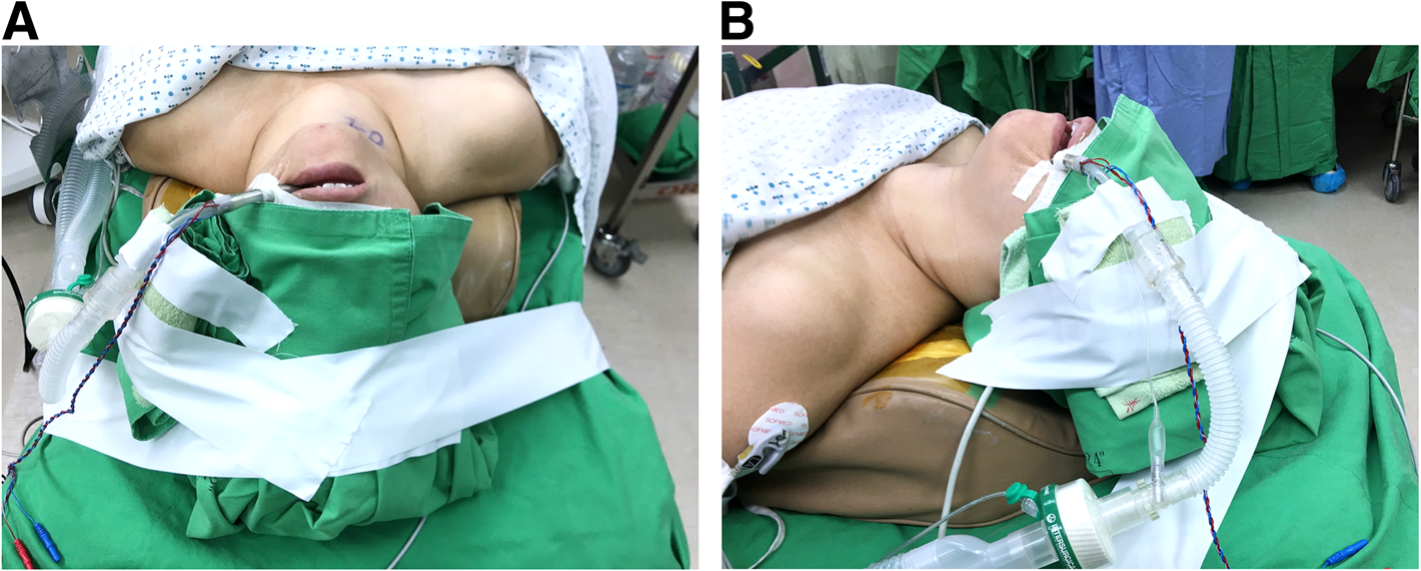
Oral endotracheal intubation with tube fixed at the mouth angle: **A**, Top view. **B**, Lateral view demonstrates the endotracheal tube running an upward course to avoid interference with lateral trocar insertion

At our institution, we routinely use oral intubation during transoral endoscopic thyroidectomy to avoid nasal complications and ensure patient comfort (Fig. 1). However, we recently encountered two cases of vocal cord granuloma that probably resulted from intraoperative compression by the oral endotracheal tube during transoral endoscopic thyroidectomy. Herein, we describe the two cases and suggest a modified position for the oral endotracheal tube to avoid such a complication.

## Case Presentation

### Case 1

A 27-year-old woman (height, 162 cm; weight, 51 kg) with a 5-year history of Graves’ disease presented with a 2.6-cm left thyroid nodule during her regular follow-up. Fine-needle aspiration cytologic examination suggested the nodule was a papillary carcinoma. She had no known history of gastroesophageal reflux disease or other systemic diseases, and her job did not require excess voice usage.

She underwent transoral endoscopic total thyroidectomy with central neck lymph node dissection. General anesthesia with oral intubation was performed using an electromyogram (EMG) tube (internal diameter = 6.0 mm) (Medtronic, Jacksonville, FL, USA) for intraoperative neuromonitoring (IONM). The endotracheal tube was fixed at the mouth angle (Fig. 1). Transoral endoscopic thyroidectomy was performed according to the procedure described by Anuwong et al. [10]. During the surgery, both the recurrent laryngeal nerves were identified visually, and their function was confirmed via IONM. The patient’s postoperative course was uneventful except for occurrence of transient hypoparathyroidism, which resolved 1 week later. Her voice was fine without any hoarseness, and she was discharged on postoperative day 4. The final pathologic examination confirmed the diagnosis of papillary carcinoma with lymph node metastasis.

**Fig. 2:**
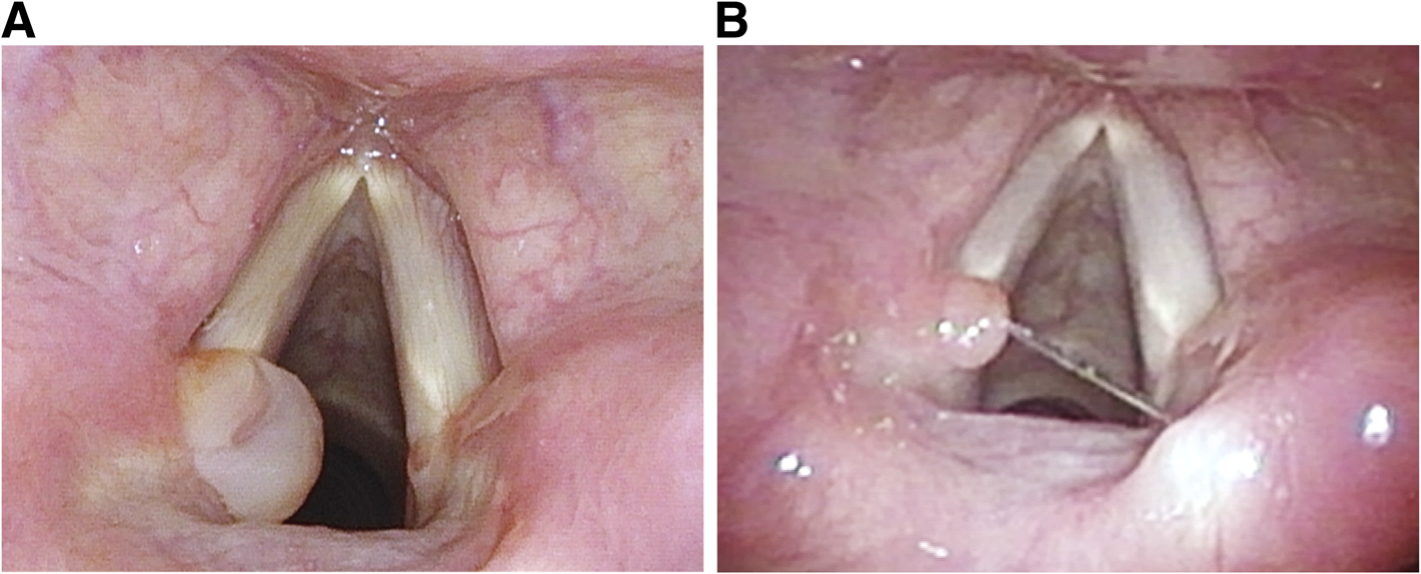
Vocal cord granuloma in case 1: **A**, Granuloma formation over the left vocal cord process was identified 2 months after transoral total thyroidectomy. **B**, After medical treatment and voice therapy, the granuloma shrank significantly 1 month later

Her condition was unremarkable during the postoperative outpatient follow-ups at 1 week and 1 month. However, she developed hoarseness, forced voice, and voice fatigue two months after the surgery. Strobolaryngoscopy revealed symmetrical and movable vocal cords but also showed a granuloma over the left vocal process (Fig. 2A). She was prescribed oral prednisolone and a proton pump inhibitor along with voice therapy. Her symptoms improved thereafter, and the granuloma appeared to have shrunk significantly as seen on the 1-month follow-up strobolaryngoscopy (Fig. 2B).

### Case 2

A 47-year-old woman (height, 168 cm; weight, 80 kg) presented with a 3.3-cm right thyroid nodule with accompanying mild compression symptom. Fine-needle aspiration cytologic examinations were performed two times, but both failed to reveal a diagnosis.

She underwent transoral endoscopic right thyroidectomy under general anesthesia with oral endotracheal intubation (tube internal diameter = 7.0 mm). IONM was also implemented and showed a positive signal from the recurrent laryngeal nerve. After the surgery, the patient regained an intact voice and showed a smooth recovery. She was discharged on postoperative day 3. At the 1-week follow-up, her condition remained unremarkable, but a pathologic examination revealed nodular goiter.

**Fig. 3:**
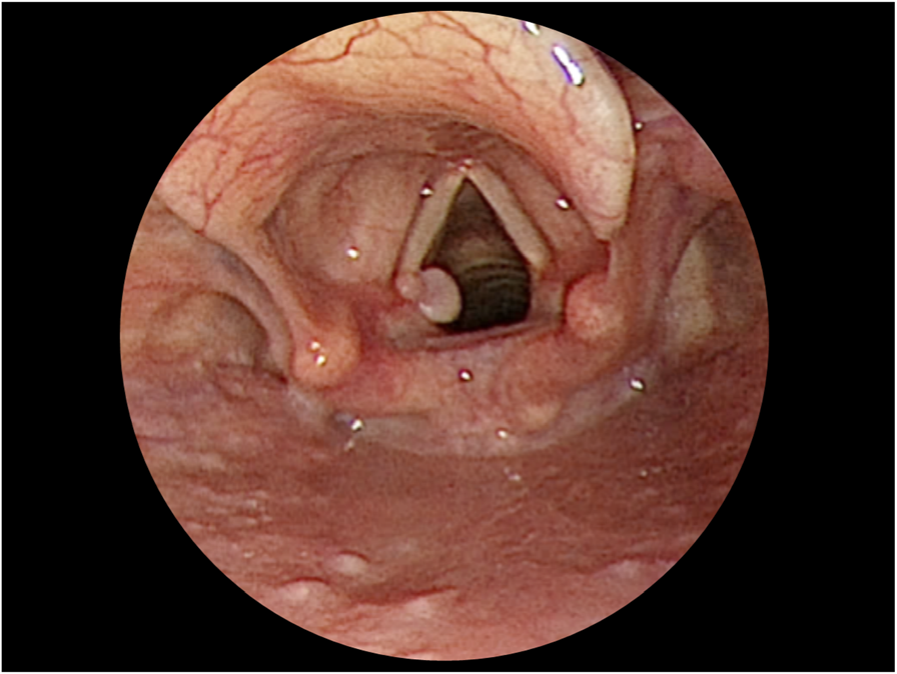
Vocal cord granuloma in case 2: A granuloma over the posteromedial aspect of the left vocal cord was noted 2 months after transoral right thyroidectomy. The location of the granuloma was similar to that of the granuloma in case 1

Nevertheless, 2 months after the surgery, the patient started noticing hoarseness with voice fatigue. Strobolaryngoscopy revealed a contact granuloma over the posteromedial aspect of the left vocal cord (Fig. 3). She denied any voice abuse and had no history of gastroesophageal reflux disease. Although she was recommended conservative treatment with medication, she opted for granuloma excision. Her symptoms improved after the surgery.

## Discussion and conclusions

To the best of our knowledge, the occurrence of vocal cord granuloma after transoral thyroidectomy has not been reported in the literature. Among 70 consecutive patients who underwent transoral endoscopic thyroidectomy at our institution, two patients (2.9 %) developed vocal cord granuloma. Their initial postoperative recovery was uneventful, but they developed hoarseness 2 months later. Further examination revealed vocal cord granulomas located in the posterior aspect of the left vocal cord in both the patients.

We speculated that granuloma formation was related to intubation trauma. We had routinely fixed the oral endotracheal tube at the patient’s left mouth angle during the surgery (Fig. 1). The connecting tube would run in a slightly upward direction to avoid collision with the left lateral trocar and then link to the anesthesia ventilator, which was placed on the patient’s left side. In this setting, the posterior part of the left vocal cord would bear the most pressure, thereby, increasing the risk of granuloma formation on that side.

Prior to surgery, we routinely performed videolaryngoscopy to check if the electrode on the EMG tube was appropriately in contact with the vocal cord after the patient was intubated and placed in the neck extension position. In both the present cases, the patients showed no lesions on their vocal cord during this laryngoscopic inspection. Moreover, they had no history of gastroesophageal reflux disease, and their jobs did not require excessive voice usage. Therefore, the vocal cord injury was more likely to have occurred during the surgery, not before it.

Other intraoperative factors that might be associated with granuloma formation in our cases included longer operative time (290 and 240 min for case 1 and case 2, respectively) and tracheal irritation caused by surgical manipulation, especially during thyroid dissection and specimen retrieval.

**Fig. 4:**
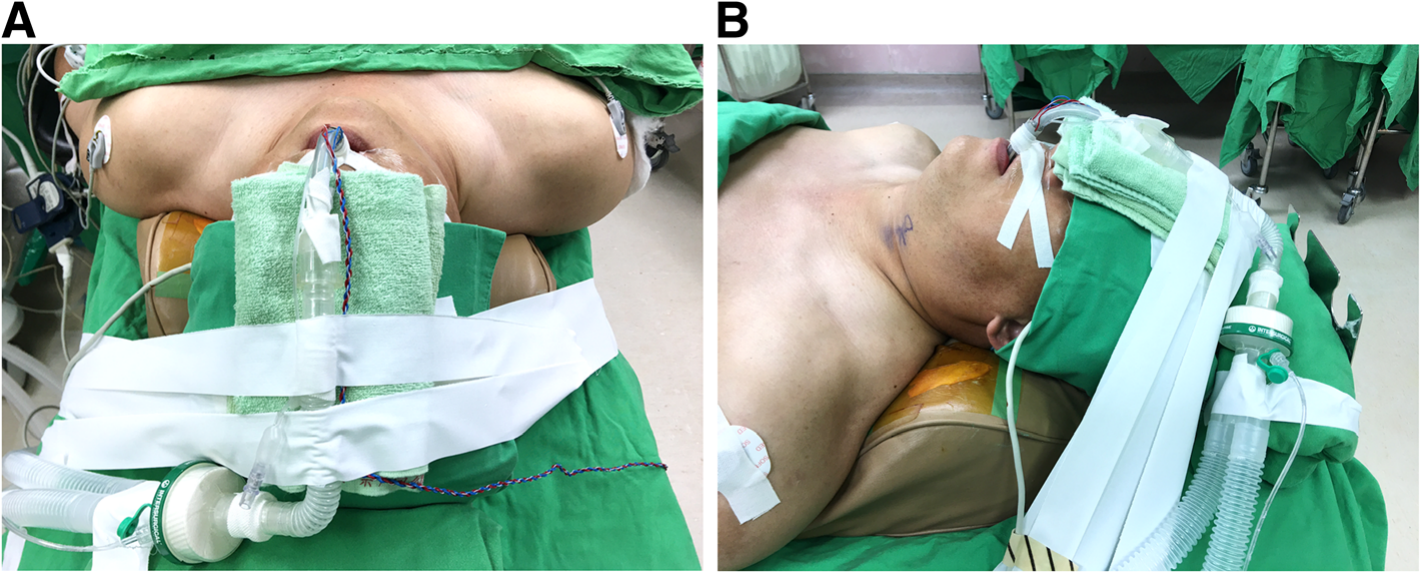
Oral endotracheal intubation with tube fixed at the midline: **A**, Top view. **B**, Lateral view demonstrates the endotracheal tube runs a curved course and crosses above the nose and forehead

After encountering these two cases, we decided to move the fixation site of the endotracheal tube from the mouth angle to the midline (Fig. 4). This change in position would enable the compression pressure of the endotracheal tube to be distributed on both sides of the vocal cord, and not solely borne on one side, which might lower the risk of granuloma formation. Care was also taken to properly fix the endotracheal tube along its natural curvature without excessive bending. After implementing this modification, we encountered no more cases of vocal cord granuloma among patients undergoing transoral endoscopic thyroidectomy.

Chai et al. also advocated for midline placement of the oral endotracheal tube to improve the movement of endoscopic instruments on the tube side of the lateral working port [11]. They reported that there was no limitation on the range of motion after changing the tube position [11].

Using nasal endotracheal intubation during transoral thyroidectomy may be another way to avoid vocal cord granuloma formation. However, care should be taken to prevent nasal complications. For example, epistaxis may be avoided by softening the endotracheal tube prior to intubation. In patients with a deviated nasal septum, the larger nostril should be used for tube insertion. The endotracheal tube should be properly positioned to avoid pressure sores on the nasal ala.

**Fig. 5:**
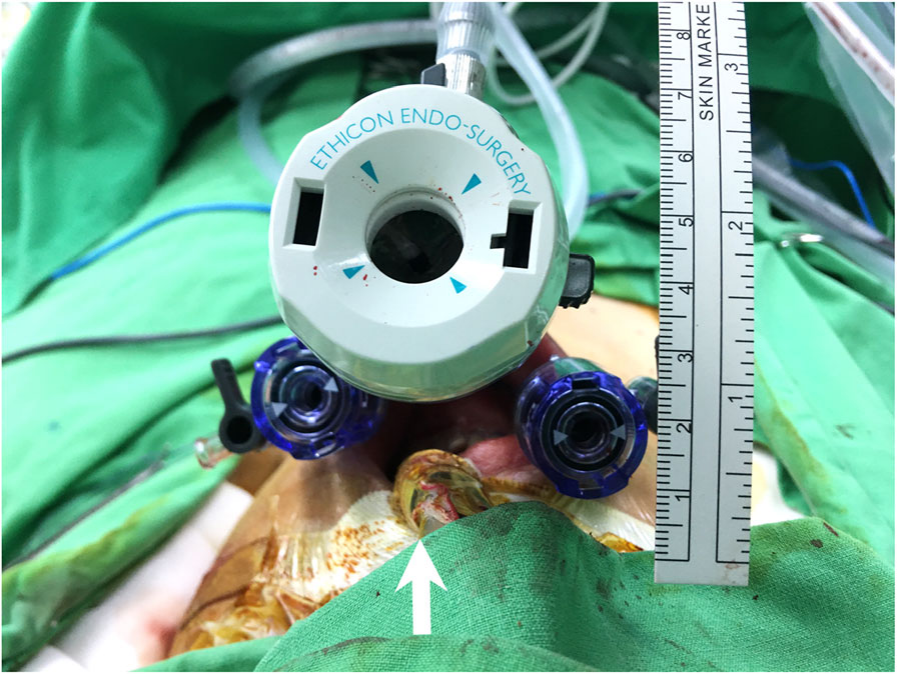
Position of the trocars and oral endotracheal tube. The middle camera port stays at a higher position than that of the oral endotracheal tube (arrow). In addition, the camera port is lifted and tilted down during the surgery so that the endoscope can be used to look down from the chin to the neck. Therefore, the chance of collision between the endoscope and the oral endotracheal tube is minimal

Some surgeons might wonder whether the midline placement of the endotracheal tube would hinder the movement of the middle trocar, which is used for inserting a 30° endoscope. In fact, the camera port stays at a higher position than the one the endotracheal tube is at (Fig. 5). Furthermore, the camera port has to be lifted and tilted down during the surgery so that the endoscope can be used to look down from the chin to the neck. Therefore, the chance of collision between the endoscope and the oral endotracheal tube is minimal. If this is still a concern, a surgeon can also use the armored endotracheal tube, which is more flexible and easier to bend to fit the curvature of the nose and forehead. This would also ensure the orotracheal tube is further away from the camera port. Another option is to fix the endotracheal tube in the paramedian position between the central camera port and the lateral working port.

Laryngeal injury due to direct pressure exerted by the endotracheal tube may result in mucosal ulceration and inflammation that lead to granuloma formation [12]. Such granulomas might initially be clinically silent but might become symptomatic weeks later [13]. Both our patients developed hoarseness 2 months after the thyroidectomy. The management of vocal cord granuloma was mainly conservative and included irritation removal and medication, which is the recommended course of treatment [12, 14]. Surgical excision is rarely required and is reserved for severe cases [12]. Our first patient showed a significant improvement in symptoms, and her granuloma shrunk after conservative treatment. However, the second patient opted for surgical excision, and she also showed substantial improvement.

In summary, we reported two cases of vocal cord granuloma resulting from compression by the oral endotracheal tube during transoral endoscopic thyroidectomy. Positioning the tube at the midline rather than at the mouth angle might decrease the risk of granuloma formation.

## Data Availability

Data sharing is not applicable to this article as no datasets were generated or analyzed during the current study.

## Abbreviations

IONM: Intraoperative neuromonitoring
